# Extended prone positioning duration for COVID-19-related ARDS: benefits and detriments

**DOI:** 10.1186/s13054-022-04081-2

**Published:** 2022-07-08

**Authors:** Thaïs Walter, Noémie Zucman, Jimmy Mullaert, Ingrid Thiry, Coralie Gernez, Damien Roux, Jean-Damien Ricard

**Affiliations:** 1 DMU ESPRIT, Service de Médecine Intensive Réanimation, AP-HP, Hôpital Louis Mourier, 92700 Colombes, France; 2 Department of Epidemiology, Biostatistics and Clinical Research, AP-HP, Hôpital Bichat, 75018 Paris, France; 3 INSERM UMR-S1151, CNRS UMR-S8253, Institut Necker Enfants Malades, Université Paris Cité, 75015 Paris, France; 4 UMR1137 IAME, INSERM, Université Paris Cité, 75018 Paris, France

**Keywords:** Prone positioning, Pressure injuries, COVID-19-related ARDS, Mechanical ventilation

## Abstract

**Background:**

During the COVID-19 pandemic, many more patients were turned prone than before, resulting in a considerable increase in workload. Whether extending duration of prone position may be beneficial has received little attention. We report here benefits and detriments of a strategy of extended prone positioning duration for COVID-19-related acute respiratory distress syndrome (ARDS).

**Methods:**

A eetrospective, monocentric, study was performed on intensive care unit patients with COVID-19-related ARDS who required tracheal intubation and who have been treated with at least one session of prone position of duration greater or equal to 24 h. When prone positioning sessions were initiated, patients were kept prone for a period that covered two nights. Data regarding the incidence of pressure injury and ventilation parameters were collected retrospectively on medical and nurse files of charts. The primary outcome was the occurrence of pressure injury of stage ≥ II during the ICU stay.

**Results:**

For the 81 patients included, the median duration of prone positioning sessions was 39 h [interquartile range (IQR) 34–42]. The cumulated incidence of stage ≥ II pressure injuries was 26% [95% CI 17–37] and 2.5% [95% CI 0.3–8.8] for stages III/IV pressure injuries. Patients were submitted to a median of 2 sessions [IQR 1–4] and for 213 (94%) prone positioning sessions, patients were turned over to supine position during daytime, i.e., between 9 AM and 6 PM. This increased duration was associated with additional increase in oxygenation after 16 h with the PaO_2_/FiO_2_ ratio increasing from 150 mmHg [IQR 121–196] at H+ 16 to 162 mmHg [IQR 124–221] before being turned back to supine (*p* = 0.017).

**Conclusion:**

In patients with extended duration of prone position up to 39 h, cumulative incidence for stage ≥ II pressure injuries was 26%, with 25%, 2.5%, and 0% for stage II, III, and IV, respectively. Oxygenation continued to increase significantly beyond the standard 16-h duration. Our results may have significant impact on intensive care unit staffing and patients’ respiratory conditions.

*Trial registration*: Institutional review board 00006477 of HUPNVS, Université Paris Cité, APHP, with the reference: CER-2021-102, obtained on October 11th 2021. Registered at Clinicaltrials (NCT05124197).

**Supplementary Information:**

The online version contains supplementary material available at 10.1186/s13054-022-04081-2.

## Background

In Europe, between May 1, 2020, and March 10, 2022, Coronavirus Disease 2019 (COVID-19) pandemic has been responsible for an estimated 270,000 hospitalizations in intensive care units (ICU) [1]. A vast majority of these patients (80%) have required tracheal intubation and invasive mechanical ventilation [2]. Whether or not acute respiratory distress syndrome (ARDS) physiopathology is the same in COVID-19 and non-COVID-19 patients has been strongly debated [3, 4]. However, a relative consensus exists on the benefits of prone position (PP) [5, 6] and 70% of patients with COVID-19-related ARDS have been turned prone [2] contrary to non-COVID-19 ARDS [7, 8].

Failure of early studies on PP to demonstrate a survival benefit in non-COVID-19 ARDS has been attributed to the insufficient duration of sessions. The first study to demonstrate a clear improvement in survival applied PP sessions of 17 h [9] and the recommended duration of PP is currently of at least 16 h [10]. Pressure injuries are the main complications associated with this technique [11, 12] and are associated with longer stays, higher costs, and higher mortality [10, 11].

Adherence to duration recommendations implies, however, that patients are turned prone between once and twice daily. Such a high frequency has several major drawbacks: intense workload, increased risk of accidental central venous catheter or tracheal tube removal at each procedure; ICU nurse and physician immobilization during the procedure; overall fatigue; viral exposition and time spent putting and removing personnel protective equipment. The high prevalence of overweight and obesity in COVID-19 patients only amplifies risks of musculoskeletal injuries for healthcare professionals.

For all these reasons, we decided since the beginning of the first wave of COVID-19 to extend the duration of PP sessions up to 48 h to minimize the number of turning procedures thus limiting staff exposure to viral contamination and reducing staff workload.

The primary objective of this retrospective observational study was to assess the safety of our strategy of extending prone position duration in a cohort of patients hospitalized for COVID-19-related ARDS by reporting the cumulative incidence of pressure injuries [15]. The secondary objectives were to determine the risk factors associated with the occurrence of pressure injuries and to quantify changes in oxygenation and ventilatory parameters during PP sessions of extended duration.

## Methods

### Study design and patients

This is an observational retrospective cohort study performed in a university-affiliated ICU from a tertiary centre where PP is routinely performed as first-line strategy during mechanical ventilation of ARDS patients with PaO_2_/FiO_2_ ratio less than or equal to 150 mmHg, whatever the aetiology [16]. Before the COVID-19 pandemic, duration of PP could be extended to a maximum of 24 h to avoid supine repositioning during nighttime. At the beginning of the COVID-19 pandemic, because of rationing of care and for practical reasons, PP duration was extended for up to a maximum of 48 h, and this new extended duration became the new standard of care. The present study was designed after the third Parisian COVID-19 surge and is thus an observational retrospective cohort study. Patients were included if they met the following criteria: being hospitalized in ICU for a PCR-proven COVID-19 infection between March 1, 2020, and April 30, 2021, having an ARDS [17] requiring tracheal intubation, having been submitted to at least one session of PP of a duration strictly greater than 24 h and age 18 years old or over. Exclusion criteria were as follows: transfer to another ICU after their initial admission in the ICU (because of ICU-bed shortage, ICU patients from the Parisian area were regularly transferred to other regions in France where the epidemic was less strong), missing information about prone position sessions or cutaneous evaluation, refusal to consent.

### Outcomes

The primary outcome was the occurrence of pressure injury of stage ≥ II during the ICU stay. Pressure injuries were defined as “a localized injury to the skin and/or underlying tissue usually over a bony prominence, as a result of pressure, or pressure in combination with shear” [18]. The occurrence of pressure injuries was evaluated retrospectively from specific pressure injury files and regular nursing charts. Gradation was made according to the Revised Pressure Injury Staging System [15] which states that a stage 1 pressure injury was defined as intact skin with a localized area of nonblanchable erythema, a stage 2 as a partial thickness loss of skin with exposed dermis, a stage 3 as a full thickness skin loss and a stage 4 as a full thickness skin and tissue loss. Secondary outcomes were the occurrence of pressure injury of stage ≥ III, the changes in oxygenation and ventilatory parameters at four different time points of the PP session: before PP session, after 16 h of PP, at the end of PP session (i.e., before turning back to supine), and after turning back to supine position.

### Exposure: protocol for PP

Patients were placed in prone position when the PaO_2_/FiO_2_ ratio was less than or equal to 150 mmHg. PP was—except in case of life-threatening hypoxemia—always initiated during daytime, and patients were kept prone for a period that covered two nights. They were put back in the supine position around 11 AM following the second night and post-PP gas were drawn one hour after being placed back into supine position. Except in case of significant respiratory deterioration, PP sessions were spaced 24 h apart (Additional file 1: Fig. S1). Prone treatment was stopped when the FiO_2_ requirement was less than or equal to 60%, when the PaO_2_/FiO_2_ was greater than 150, and when the PEEP was inferior or equal to 12 cm of H_2_O. Enteral nutrition was continued during all PP sessions, in the absence of intolerance and with an objective of 20–30 kcal/kg per day. Enteric tubes were not required to be post-pyloric prior to proning.

Concerning PP installation, the proning team included the nurse, the assistant nurse, the resident, and the fellow in charge of the patient and between one and three additional persons that could be a physiotherapist, or other residents or medical students. No mechanical assist devices were used. The patient was positioned with a thoracic cushion under their shoulders, freeing the neck. A pelvic cushion was placed at the level of the iliac bones. The head cushion was placed in such a way as to render the endotracheal tube visible and accessible and to avoid pressure points on the eyes, the nose or the chin. The arms were placed at the side of the body with the palms of the hands facing upwards. The feet protruded from the bed. The bed was positioned proclive at 20°. Every 4 h the head was repositioned and eye care was performed. The position of the patient and his installation was adapted according to size and morphology. This protocol is in accordance with the guidance of Intensive Care Society and The Faculty of Intensive Care Medicine [19].

### Protocol for the prevention and management of pressure injuries

Pressure injuries prevention methods were part of daily care and encompassed alternating-air mattresses, regular repositioning, and daily nurse skin assessment [20]. Despite this protocol, if, at any time of the ICU stay, a patient developed pressure injuries stage II or above, a specific and dedicated “pressure injuries file” was completed. Pressure injuries of stage I were not specifically monitored.

### Management of COVID-19-related ARDS

The diagnosis of COVID-19 was made by means of a PCR test performed on an oro-pharyngeal swab or an endotracheal aspirate. From July 2020 onwards, patients were systematically given corticosteroids [21] (Dexamethasone 6 mg per day for 10 days or 10 mg per day in case of body mass index > 30) in combination with anti-IL6 immunotherapy (Tocilizumab 8 mg per kg (maximal dose 800 mg). The bolus of anti-IL6 immunotherapy could be repeated 72 h later.

ARDS was managed according to the recommendations of the French intensive care society (SRLF) [10]. Patients were deeply sedated based on the Richmond agitation sedation scale (RASS) with a RASS -5 as target. During the first 48 h of ARDS, patients systematically received neuromuscular blocking agents. In cases where patients required PP after the first 48 h, neuromuscular blocking agents were maintained as long as patients required PP. Protective ventilation was provided with a tidal volume of 6–8 ml/kg of their predicted body weight [22]. The positive end expiratory pressure (PEEP) was set to a minimum of 8 cm of H_2_O with a plateau pressure less or equal to 30 cm H_2_O. In case of PaO_2_/FiO_2_ less than 80, inhaled nitric oxide (iNO) was added. In case of non-response to iNO in terms of PaO_2_, the use of extracorporeal membrane oxygenation (ECMO) was discussed.

### Data collection

Demographics data were collected from medical files. Patients with a history of either chronic heart failure, ischemic heart disease or peripheral arterial disease were grouped under the term of “history of cardiovascular disease”. Patients with a history of either chronic obstructive pulmonary disease, asthma, or chronic respiratory failure were grouped under the term of “history of respiratory disease”. Cutaneous complications were retrieved from specific “pressure injuries files” and nursing charts. Incidence of brachial plexus injury and in-hospital mortality were retrieved from medical files of the last hospital ward. Ventilatory parameters were retrieved from the daily monitoring charts and blood gas results from computerized records. For each PP session, values of PaO_2_, PaCO_2_, PaO_2_/FiO_2_ ratio, PEEP and dynamic respiratory system compliance were collected. Sessions were qualified as being “O_2_-responders” if they were associated with an increase in the PaO_2_/FiO_2_ ratio of more than 20 mmHg at H+ 16 after the beginning of the prone session [23, 24].

### Statistical analysis

Descriptive data are presented as percentage for categorical variables, and as median [25th–75th interquartile range] for quantitative variables. Differences in demographical data between the analysed patients and the excluded population were assessed with the Fisher's exact test or Wilcoxon test. The cumulative incidence of pressure injuries during the stay is estimated with its exact binomial 95% confidence interval. Factors associated with the occurrence of a pressure injury during the stay are studied using a univariate logistic model. Crude odds ratios are reported with their asymptotic 95% confidence interval and the *p* value of the Wald test. The evolution of gas exchanges parameter between time points is described with median and interquartile ranges. In order to take the correlation between responses across different PP sessions of the same patient into account, a linear mixed model is used, with the parameter as response variable and the patient as random intercept. The intercept coefficient and its standard error were estimated by minimizing the restricted maximum likelihood. The *p* value and 95% confidence interval were derived according to the Satterthwaite approximation. A *p* value inferior or equal to 0.05 was considered as statistically significant.

All analyses were done in R version 4.1.2. (The “R” Foundation for Statistical Computing, Vienna, Austria). Data that support the findings of this study are available from the corresponding author on reasonable request.

### Ethical approval

This study was approved by the Ethics Evaluation Committee of Biomedical Research Project (Comité d’Evaluation de l’Ethique des projets de Recherche Biomédicale (CEERB)) Paris Nord (institutional review board—IRB 00006477 of HUPNVS, Université Paris Cité, APHP) with the following reference CER-2021-102 and registered at Clinicaltrials (NCT05124197). Due to the retrospective design of the study, written consent was waived and the registration at Clinicaltrials was completed once we decided to report our experience and results with the extended PP strategy. Patients and families were informed of their right to decline use of their data for the research.

## Results

### Participants

**Fig. 1:**
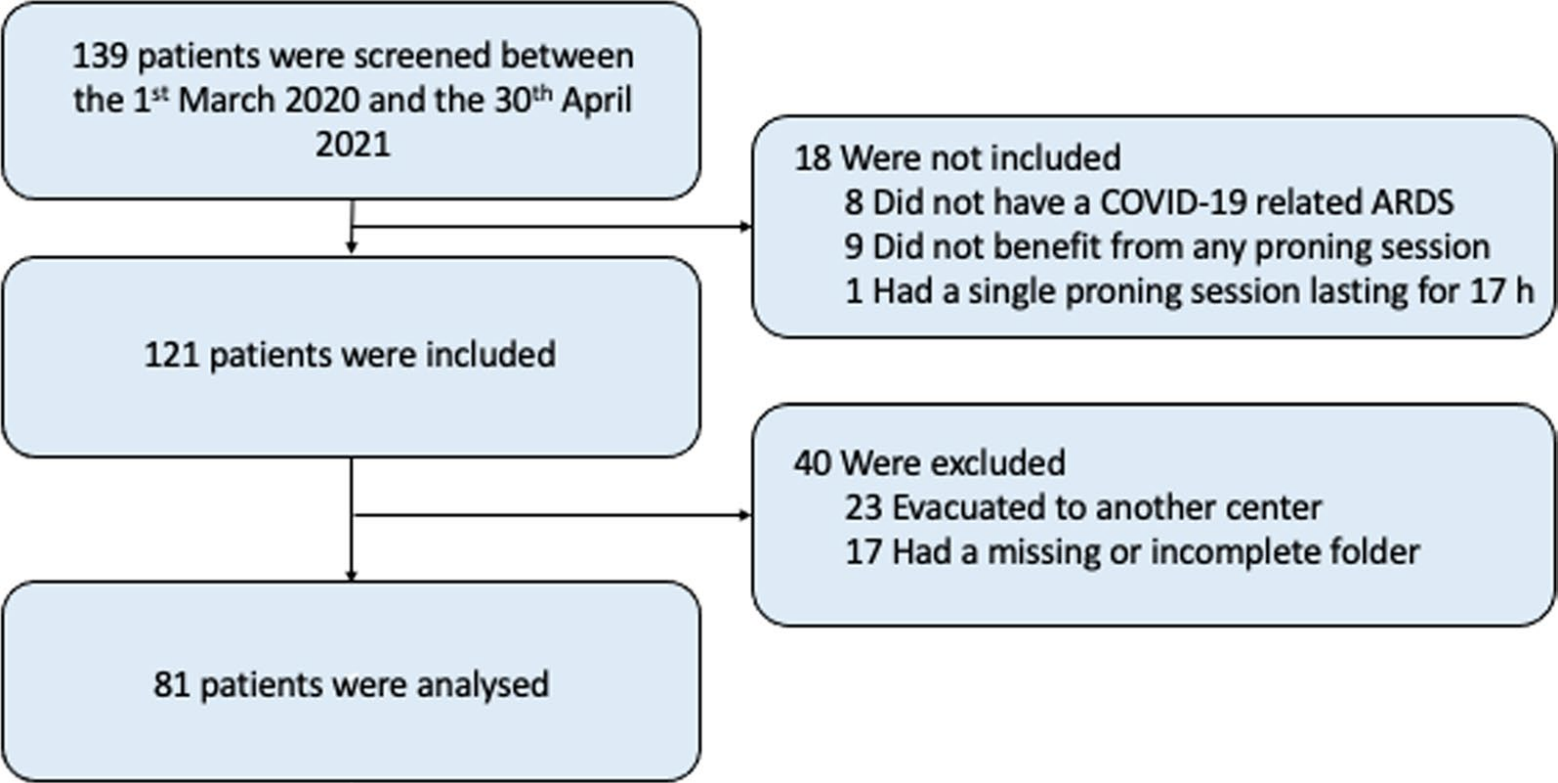
Flowchart of the study. COVID-19 = Coronavirus disease 2019, ARDS = Acute respiratory distress syndrome

Between March 1, 2020, and April 30, 2021, 121 patients were included (Fig. 1). The analysed sample was composed of 81 patients, after exclusion of 23 patients transferred to another ICU and 17 patients whose medical files were incomplete. Demographic characteristics of the analysed sample and excluded patients were similar except for the status of active smoker (Table 1). The median age was 60 (interquartile range [IQR] 51–67) years and more than half were obese (median Body Mass Index 31 [IQR 27–36], Table 1). None of them presented with pressure injury upon admission into the ICU. All had severe COVID-19-related ARDS and required PP (median PaO_2_/FiO_2_ ratio before first PP session 78 mmHg [IQR 67–100]. In addition, 34 (42%) patients received inhaled nitric oxide (42%), and 17 (21%) required the use of extracorporeal membrane oxygenation. In-hospital mortality rate was 31% (n = 25) (Table 2).

**Table 1:** Baseline characteristics of the analysed patients (*n* = 81) and the excluded patients (*n* = 40)

Baseline characteristics	Analysed patients (*n* = 81)	Excluded patients (*n* = 40)	p* for diff
Age, years	60 [51–67]	60 [52–67]	0.97
BMI, kg/m^2^ (missing = 3)	31 [27–36]	30 [28–34]	0.50
SAPS II score	38 [30–46]	38 [28–50]	0.98
Sex, male	58 (72)	24 (60)	0.22
Active smoker	13 (16)	1 (2)	**0.03**
Type 2 diabetes	22 (27)	11 (28)	0.88
History of cardiovascular disease**	8 (10)	1 (2)	0.27
History of respiratory disease***	8 (10)	4 (10)	1
Chronic kidney disease	5 (6)	4 (10)	0.48
Cirrhosis	5 (6)	0 (0)	0.17

Data are presented as No. (%) or median [interquartile range]

BMI, Body Mass Index; SAPS II, simplified acute physiology score

*Fisher exact test or Wilcoxon test; **chronic heart failure, ischemic heart disease or peripheral arterial disease; ***chronic obstructive pulmonary disease or Asthma.

Bold formatting of *p* value indicates a statistically significant value

**Table 2:** Patients’ respiratory parameters before the first prone position session, respiratory management and outcome

Variables	Analysed patients (*n* = 81)
PaO_2_/FiO_2_ ratio, mmHg (missing = 5)	78 [67–100]
PEEP, cmH_2_O	12 [10–13]
Driving pressure, cmH_2_O (missing = 13)	12 [10–14]
Respiratory system compliance, mL/cmH_2_O (missing = 13)	34 [27–43]
ICU length of stay, days	19 [12–30]
Duration of mechanical ventilation, days	21 [9–30]
Duration of proning sessions, h	39 [34–42]
Number of proning sessions	2 [1–4]
Continuous neuromuscular blockers	81 (100)
ECMO	17 (21)
Nitric oxide	34 (42)
Renal replacement therapy	5 (6)
ICU mortality	24 (30)
At least one pressure injury	21 (26)
At least one pressure injury of stage III or IV	2 (2)
At least one proning session with catecholamines	37 (46)

Data are presented as No. (%) or median [interquartile range]

PaO_2_, partial pressure of dioxide in arterial blood; PaCO_2_, partial pressure of carbon dioxide in arterial blood; PEEP, Positive End Expiratory Pressure; ICU, intensive care unit; ECMO, Extracorporeal Membrane Oxygenation

Bold formatting of *p* value indicates a statistically significant value

### Prone positioning sessions

A total of 227 PP sessions were performed during the study period. Patients had a median of 2 [IQR 1–4] sessions that lasted for a median of 39 h [IQR 34 – 42]. Supine repositioning was planned in the vast majority of cases (n = 201, 89%). Unplanned supine repositioning were mostly secondary to the death of the patient (n = 7, 3%) or the need for an ECMO cannulation (n = 5, 2%) (Table 3). Only 2 (0.9%) PP sessions were terminated early because of cutaneous complications. PP sessions with an unplanned turned to supine had a median duration of 25.25 h [IQR 18.00–28.75]. For 213 (94%) PP sessions, patients were turned over to supine position during daytime, i.e., between 9 AM and 6 PM. In comparison, had PP sessions lasted strictly for 16 h, half of them (n = 117, 52%) would have required a turning back to supine between 6 PM and 9 AM, i.e., during night shifts. Of the 227 PP sessions, 2 (0.9%) were performed without continuous neuromuscular blockers and 13 (6%) were performed under veno-venous-ECMO. Enteral feeding could be continued throughout 154 (68%) sessions. No unscheduled extubation nor dislodging of catheters (including ECMO cannula) was reported. One patient (1.2%) suffered from a brachial plexus injury that fully recovered.

**Table 3:** Reported reasons for ending prone position sessions

Reasons for turning back to supine	All prone position sessions (*n* = 227)
Planned	201 (89)
Death while in prone position	7 (3)
No response in terms of increase in the PaO_2_/FiO_2_ ratio	5 (2)
Indication for ECMO	5 (2)
Other	3 (1)
Cutaneous complications	2 (1)
Tracheal tube obstruction	2 (1)
Computed tomography	1 (0)
No reason reported	1 (0)

Data are presented as No. (%)

PaO_2_, partial pressure of dioxide in arterial blood; FiO_2_, fraction of inspired oxygen; ECMO, Extracorporeal Membrane Oxygenation

Bold formatting of *p* value indicates a statistically significant value

For 51 sessions, patients did not tolerate being left supine and PP was resumed before 11 AM the next morning.

### Pressure injuries and associated factors

The cumulative incidence of pressure injuries during the stay was 26% (95% CI 17–37) for stage ≥ II pressure injuries and 2.5% (95% CI 0.3–8.8) for stages III/IV pressure injuries. Out of the 43 recorded pressure injuries, 42 (98%) were located on the ventral side of the body (Additional file 2: Table S1). The number of stages II, III, and IV pressure injuries was 41, 2, and 0 respectively. The two main factors associated with the occurrence of pressure injuries were the cumulative duration of PP sessions (OR 1.33, 95% CI 1.11–1.63 per 24 h, *p* = 0.0015) and the ICU length of stay (OR 1.05, 95% CI 1.01–1.10 per additional day, *p* = 0.021) (Table 4). The duration of each session was not associated with pressure injury occurrence. None of the stage III/IV injuries required surgical debridement.

**Table 4:** Factors associated with the occurrence of pressure injuries (univariate logistic regression)

Variables	Patients without pressure injury (*n* = 60) *n*/*N* (%) or med (Q1–Q3)	Patients with at least one pressure injury (*n* = 21) *n*/*N* (%) or med (Q1–Q3)	OR (95% CI)	*p*
Sex, male	40/60 (67)	18/21 (86)	3.00 (0.88–13.88)	0.081
Age, years	60 (51–68)	58 (52–63)	1.00 (0.96–1.05)	0.91
BMI, kg/m^2^	30 (27–37)	33 (29–36)	0.99 (0.94–1.01)	0.54
Type 2 diabetes	13/60 (22)	7/21 (33)	1.43 (0.50–3.88)	0.49
History of cardiovascular disease*	7/60 (12)	1/21 (5)	0.38 (0.02–2.32)	0.33
History of respiratory disease**	6/60 (10)	2/21 (10)	0.95 (0.13–4.53)	0.95
SAPS II score	36 (30–48)	39 (33–42)	0.99 (0.95–1.02)	0.43
PaO_2_/FiO_2_ ratio before first pronation session, mmHg	82 (68–114)	88 (75–99)	1.00 (0.98–1.01)	0.65
ECMO	12/60 (20)	5/21 (24)	1.25 (0.35–3.96)	0.71
Nitric oxide	25/60 (42)	9/21 (43)	1.05 (0.38–2.86)	0.92
At least one proning session with catecholamines	28/60 (47)	9/21 (43)	0.86 (0.31–2.33)	0.76
Duration of mechanical ventilation, days	14 (9–25)	29 (22–34)	1.01 (1.00–1.02)	0.12
ICU length of stay, days	17 (11–26)	29 (19–34)	1.05 (1.01–1.10)	**0.021**
Median duration of each proning sessions, h	40 (35–43)	36 (32–40)	0.96 (0.89–1.03)	0.26
Cumulated duration of all proning sessions, days^***^	3 (2–5)	6 (4–8)	1.33 (1.11–1.63)	**0.0015**

BMI, Body Mass Index; SAPS II, simplified acute physiology score; ECMO, Extracorporeal Membrane Oxygenation; PaO_2_, partial pressure of dioxide in arterial blood; FiO_2_, fraction of inspired oxygen; ICU, intensive care unit; PP, prone positioning

***This duration is the added duration of all PP sessions for each patient *chronic heart failure, ischemic heart disease or peripheral arterial disease; **chronic obstructive pulmonary disease or Asthma

Bold formatting of *p* value indicates a statistically significant value

### Evolution of oxygenation and ventilatory parameters

**Fig. 2:**
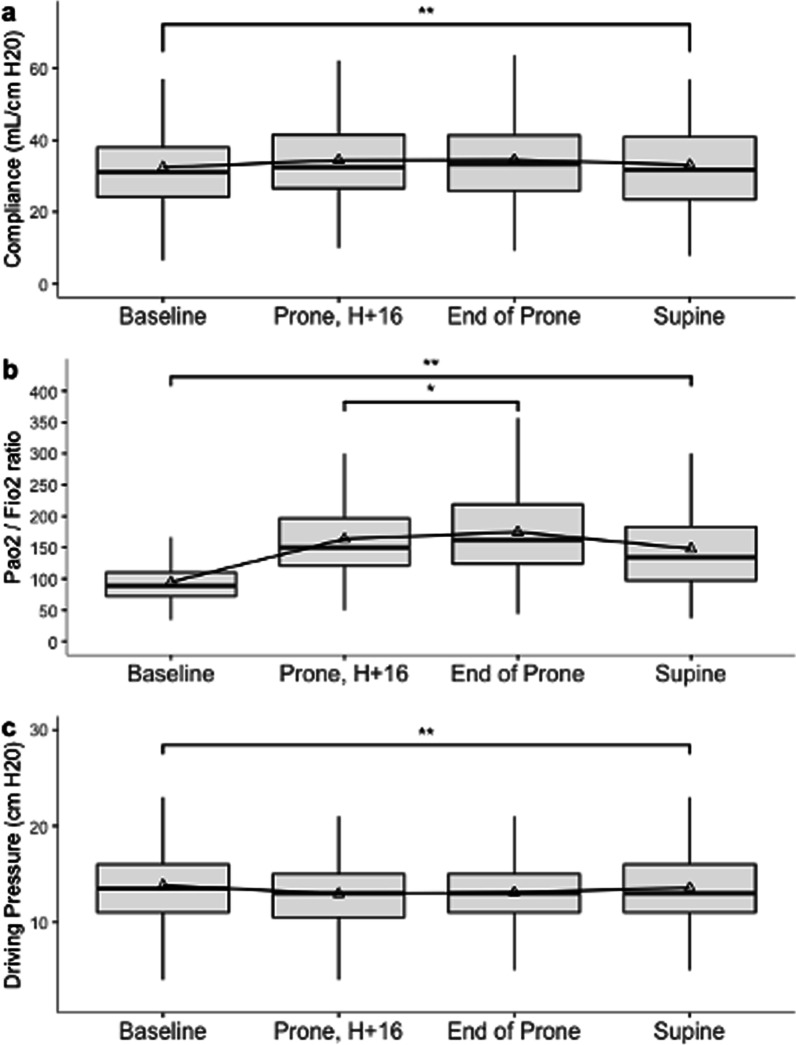
Changes in physiological parameters. (**a** Compliance,** b** PaO_2_/FiO_2_, **c** driving pressure) during proning sessions

PP was associated with significant increase in oxygenation and decrease in PaCO_2_ between pre-PP session and after turning back to supine (Additional file 2: Table S2). Notably, PaO_2_/FiO_2_ also increased significantly during PP (Fig. 2). Moreover, the increase of PaO_2_/FiO_2_ ratio, between pre-PP and after turning back to supine of the first PP session, was associated with a reduced ICU mortality (OR 0.89, 95% CI 0.83–0.97 per 10 mmHg increase, *p* < 0.01).

To assess the additional benefit in terms of improved oxygenation of our strategy over that of a 16-h session, we compared oxygenation parameters measured around the 16th hour of PP and those measured at the end of the extended PP session (i.e., before turning back to supine). In this period (Fig. 2), the mean increase of the PaO_2_/FiO_2_ ratio was 60 mmHg (95% CI 47–73, *p* < 0.01) and the mean pH increase was 0.01 (95% 0–0.02, *p* = 0.013). These improvements between the 16th hour of PP and the end of the extended PP session, for both the PaO_2_/FiO_2_ ratio and the pH, were also found in a sensitivity analysis performed only on the first PP sessions (Additional file 2: Table S3).

**Fig. 3:**
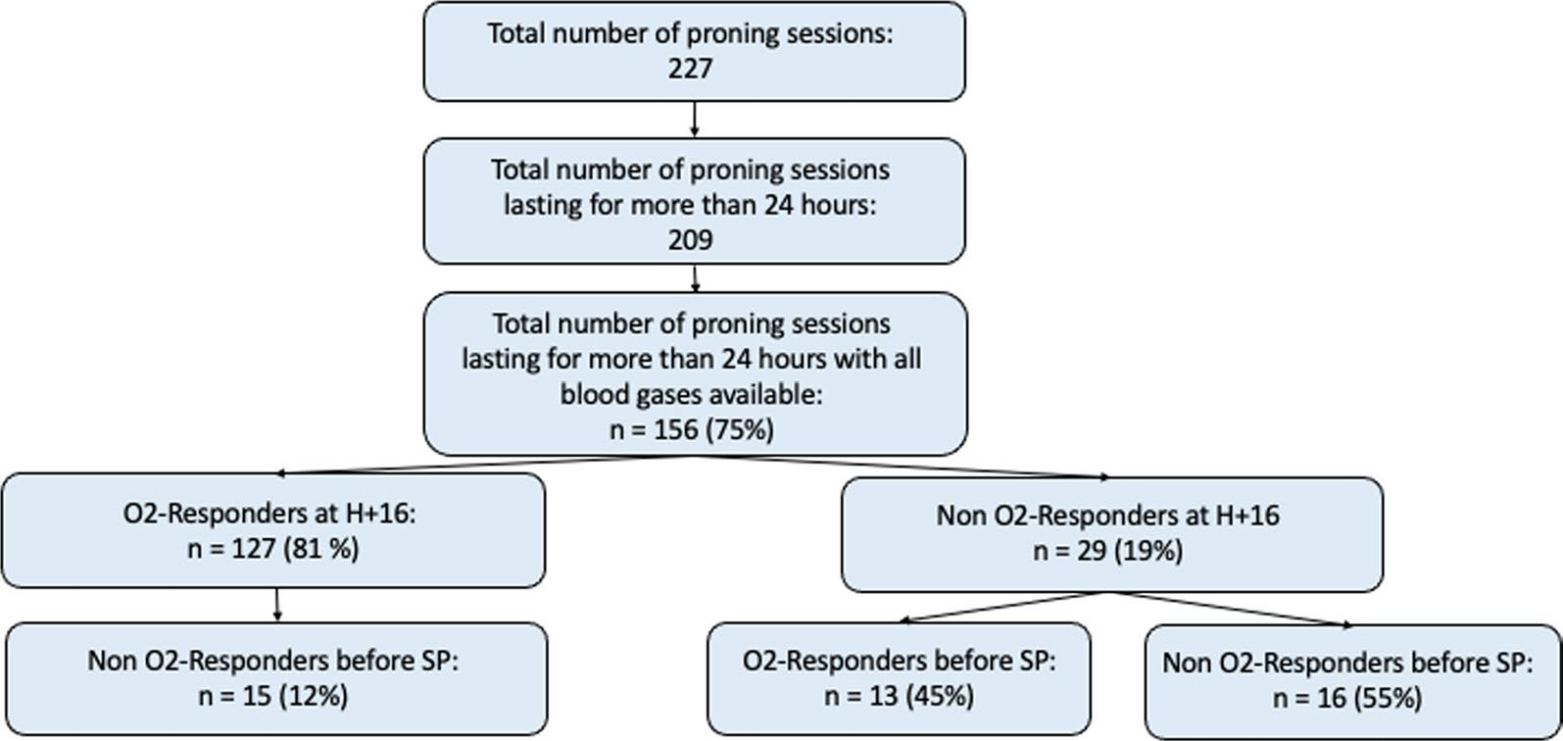
Flowchart O_2_-Responders during extended prone position sessions. O_2_-Responders = session with an increase of + 20 mmHg in the PaO_2_/FiO_2_ ratio. H = hour; n = number; SP = supine position

During PP sessions which lasted more than 24 h and for which all blood gases were available, 81% (n = 127) were O_2_-Responder sessions at H+ 16. Among non-O_2_-Responder sessions at H+ 16, 45% (n = 13) became O_2_-Responder on the blood gas made before being turned back to supine (Fig. 3). Finally, between pre-PP and after turning back to supine, FiO_2_ was lowered and the dynamic respiratory system compliance improved (Additional file 2: Table S2).

## Discussion

In this monocentric retrospective cohort study of patients with COVID-19-related ARDS requiring PP, we found that a strategy of extending duration of PP sessions for a median of 39 h, was safe and resulted in a cumulative incidence of stage ≥ II pressure injury of 26%, a very low rate of stages III and IV pressure injuries, a reduced number of PP sessions and turnings back to supine occurring during nighttime, and was associated with additional increase in oxygenation after 16 h. Given the number of patients requiring PP because of COVID-19-related ARDS, our results may have significant impact on both ICU staffing and organizational issues and could improve ventilatory parameters.

Pressure injuries are the most common complications of PP. In an ancillary study of an international randomized trial evaluating PP in ARDS, a cumulated incidence of 25% of patients presenting for the first time a pressure injury of stage ≥ II between day 1 and ICU discharge was reported [11]. This is in line with our findings of a cumulated incidence of 26%. Other studies also included stage I in their cumulated incidence pressure injuries and report between 41 and 46% [12, 25]. This inclusion of stage I pressure injury may not be that relevant and renders the comparison with the present study difficult. Pressure injuries of stage superior or equal to III remained rare events in our strategy with a cumulated incidence of 2.5% (0.3–8.8). The only other study reporting the cumulated incidence of high-stage pressure injuries is a prospective study in which COVID-19 ARDS patients were prone for a median of 3 days (IQR = 2–5 days). The associated cumulated incidence of stage IV pressure injury was 3.3% but they were all of supine (sacral) localization, which seems counterintuitive. Importantly, the cumulated incidence of patients presenting a stage ≥ I pressure injury for the first time was 70.5% which was much greater than previous results [11]. In that study 8.2% of patients developed a brachial plexus injury which is in line with the incidence of 7/114 (6%) in a cohort of COVID-19 ARDS patients [26]. Both of these incidences are much greater than the one we report (n = 1, 1.2%). Taken together, these results suggest that extending PP sessions to 48 h does not seem to increase the cumulated incidence of pressure injuries, while extending sessions beyond 48 h may considerably increase this incidence.

In various studies, reported duration of PP sessions are around 18 h [8, 9, 12, 25, 27, 28]. The strategy we adopted in COVID-19-related ARDS resulted in twice as long PP sessions (39 h). This extended duration allowed patients to be turned back to supine during daytime for 94% of the sessions. Had the 16-h duration been strictly applied to our cohort, as in some ICUs [28], then more than half of the turnings back to supine would have occurred during night shift periods, when medical staffing level is reduced. Because night shifts have been associated with more adverse events such as unplanned extubation [29] and mechanical complications of central venous lines insertion [30] and as these turning manoeuvres may be associated with adverse events (incidence of accidental extubation ranges from 0.9 to 1.25% [9, 31]), it seems preferable to perform them when more physicians are available. The median number of PP sessions was also significantly lower in the present study than in other observational studies [25, 27, 28]. The maximum number of PP sessions for a given patient in the present study was 8, for a cumulative PP duration of 350 h. Had the 16-h duration been strictly implemented, this patient would have potentially been turned prone 22 times. This patient presented a single injury, located at the corner of the mouth that heeled uneventfully.

Many more patients were turned prone during the pandemic than before, resulting in a considerable increase in workload and leading some to create and train dedicated teams of non-ICU physicians to perform PP [32]. As these turning manoeuvres are not without risks as mentioned above, having to perform a large number of manoeuvres runs the risk of increasing occurrences of complications, especially when taken care of by non-specialized staff. Our strategy enables to considerably reduce the number of manoeuvres and hence the healthcare workers’ exposure to high physical demand with poor associated posture, especially given the high prevalence of obesity in patients with COVID-19-related ARDS. This can be beneficial given the high prevalence of musculoskeletal injuries among ICU nurses [33] and its association with physical demand [34]. In addition, each manoeuvre exposes between 5 and 7 healthcare workers to the risk of viral contamination. Again, our strategy reduces unnecessary viral exposure.

The improvement in oxygenation and ventilatory parameters we observed are in line with those reported in other studies with a significant increase of the PaO_2_/FiO_2_ ratio between pre-PP and post-supine positioning [25, 27, 28, 35]. Additionally, we show a significant increase in the dynamic compliance between before proning sessions and after proning sessions. Although the improvement in static compliance have been well shown in PP for non-COVID-19-related ARDS [9, 36, 37], it had not yet been shown for COVID-19-related ARDS [27, 28]. The improvement of the PaO_2_/FiO_2_ ratio was further studied by dichotomizing the evolution observed between “O_2_ responders” and “non-O_2_ responders”. In the present cohort, at H+ 16 after being turned prone, patients were O_2_-responders in 81% of the sessions. Waiting until 11 AM the next morning before turning patients back on supine position was associated with patients reaching the criteria of being an O_2_ responder during an extra 8% of sessions. As in other studies concerning PP in COVID-19 ARDS [27, 28], we have found a significant association between an improved oxygenation and a reduced mortality. This contrasts with an ancillary study of the PROSEVA study [9, 24] where no association was found. However, due to the retrospective nature of the present study and its small sample size, no causal inference can be made and the association must be interpreted with caution.

It was not possible to have a control group in terms of pressure injuries owing to the design of the study for the following reasons. First, this was not an interventional study. The decision to extend duration of prone position was established as part of our routine practice since the start of the pandemic and was hence applied to all patients during all the surges. There were no historical controls either, because of the novelty of COVID-19. Finally, regarding oxygenation and ventilation parameters, patients were their own controls, since we evaluated the persistence or not, of oxygenation improvement beyond the “usual” 16–18-h duration of prone position.

Other limitations are inherent to the retrospective design of the study. Namely, the incidence of pressure injuries may have been underreported and thus underestimated. Although this might be true for non-serious injuries (stage I or II) (even though the figures we report are in line with those in the literature), we believe this was not the case for the more serious injuries. Indeed, because our strategy to extend the duration was newly implemented, staff was regularly reminded to pay a particular attention to potential complications of this strategy. In addition, nurses were questioned during morning rounds on the tolerance of the PP specifically in terms of occurrence of pressure injuries, and a dedicated form to report and monitor stage ≥ II injuries was implemented.

Finally, patients transferred to another ICU after their initial admission in the ICU were excluded from the analysis, leading to a possible bias in the estimation the cumulative incidence of pressure injuries. However, looking at baseline characteristics, risks of pressure injuries did not seem to be different between transferred and non-transferred patients. Indeed, only the active smoking status was more prevalent in the analysed cohort, possibly leading to an overestimation of the cumulated incidence of pressure injury.

Whether this strategy should be applied to non-COVID-19-related ARDS patients remains to be determined. In particular, our strategy may not be applicable to patients with severe septic shock-related ARDS because of cutaneous perfusion alterations [38].

## Conclusions

A strategy based on extending duration of proning sessions up to 48 h in patients ventilated for COVID-19-related ARDS, originally implemented for organizational and human resources purposes, did not seem to increase the risk of pressure injuries compared with PP sessions of shorter duration in non-COVID-19-related ARDS. It significantly reduced staff workload and viral exposure and was associated with further improvements in oxygenation and ventilatory parameters during the prone sessions.

## Supplementary Information

**Additional file 1: Figure S1.** Protocol for proning. PP = prone positioning, SP = supine positioning, P/F = PaO_2_/FiO_2_ ratio.

**Additional file 2: Table S1.** Anatomical localization of pressure injuries. **Table S2.** Ventilatory parameters during proning sessions (*n* = 227 sessions). **Table S3.** Ventilatory parameters during the first proning session (*n* = 81 sessions)

## Data Availability

The datasets analysed during the current study are not publicly available due to the risk of compromising individual privacy but are available from the corresponding authors on reasonable request.

## Abbreviations

ARDS: Acute respiratory distress syndrome
CI: Confidence interval
COVID19: Coronavirus disease 2019
ECMO: Extracorporeal membrane oxygenation
ICU: Intensive care units
IQR: Interquartile range
iNO: Inhaled nitric oxide
OR: Odd ratio
PCR: Polymerase chain reaction
PEEP: Positive end expiratory pressure
PP: Prone positioning
RASS: Richmond agitation Sedation Scale
