# Supplementary material for: Extended prone positioning duration for COVID-19-related ARDS: benefits and detriments
Source: Crit Care. 2022 Jul 8;26:208. doi: 10.1186/s13054-022-04081-2 (PMC9263064; doi:10.1186/s13054-022-04081-2)
Supplement: Supplementary file 1 — Additional file 1: Figure S1. Protocol for proning. PP = prone positioning, SP = supine positioning, P/F = PaO2/FiO2 ratio. [file 13054_2022_4081_MOESM1_ESM.pptx]

## Slide 1
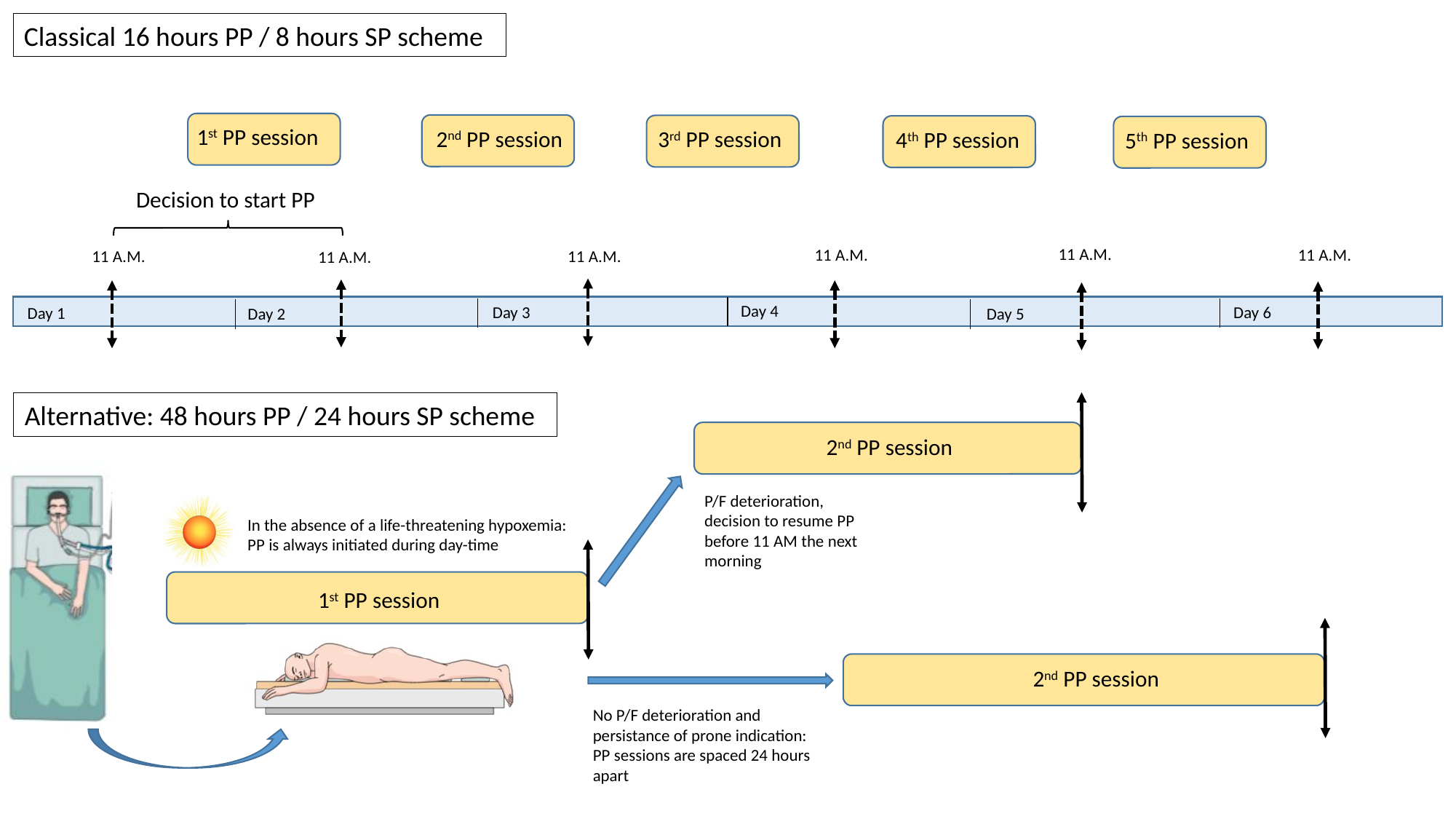

Classical 16 hours PP / 8 hours SP scheme
1st PP session
2nd PP session
3rd PP session
4th PP session
5th PP session
Decision to start PP
11 A.M.
11 A.M.
11 A.M.
11 A.M.
11 A.M.
11 A.M.
Day 4
Day 3
Day 6
Day 1
Day 2
Day 5
Alternative: 48 hours PP / 24 hours SP scheme
2nd PP session
P/F deterioration, decision to resume PP before 11 AM the next morning
In the absence of a life-threatening hypoxemia:
PP is always initiated during day-time
1st PP session
2nd PP session
No P/F deterioration and persistance of prone indication:
PP sessions are spaced 24 hours apart
