# Supplementary material for: Extended prone positioning duration for COVID-19-related ARDS: benefits and detriments
Source: Crit Care. 2022 Jul 8;26:208. doi: 10.1186/s13054-022-04081-2 (PMC9263064; doi:10.1186/s13054-022-04081-2)
Supplement: Supplementary file 2 — Additional file 2: Table S1. Anatomical localization of pressure injuries. Table S2. Ventilatory parameters during proning sessions (n = 227 sessions). Table S3. Ventilatory parameters during the first proning session (n = 81 sessions) [file 13054_2022_4081_MOESM2_ESM.docx]

| **SUPPLEMENTARY TABLE S1** – Anatomical localization of pressure injuries   \| **Anatomical sites** \| **Number of pressure injuries (N=43),  n (%)** \| \| --- \| --- \| \| Labial commissure / Mouth \| 14 (32.6) \| \| Face, outside the mouth \| 9 (20.9) \| \| External genitalia \| 8 (18.6) \| \| Abdominal / Thorax \| 3 (7) \| \| Feet \| 2 (4.7) \| \| Lower limbs \| 2 (4.7) \| \| Sacral \| 2 (4.7) \| \| Shoulders \| 2 (4.7) \| \| Tracheostomy \| 1 (2.3) \|   Data are presented as number (percentage). N indicates the total number of pressure injuries among the whole cohort. n = number of pressure injuries at the given anatomical site. |
| --- | --- | --- | --- | --- | --- | --- | --- | --- | --- | --- | --- | --- | --- | --- | --- | --- | --- | --- | --- | --- |

**SUPPLEMENTARY TABLE S2 –** Ventilatory parameters during proning sessions (n = 227 sessions)

| **Variables** | **Before PP** | **At 16 hours of PP** | **End of PP** | **After SP** | **p for evolution between before PP and after SP** | **p for evolution between 16 hours of PP and end of PP** |
| --- | --- | --- | --- | --- | --- | --- |
| Dynamic respiratory system compliance, mL/cmH_2_O | 32 [24-40] | 33 [27-43] | 34 [26-43] | 34 [24-42] | **0.014** | 0.954 |
| Driving pressure, cmH_2_O | 14 [11-16] | 13 [10-15] | 13 [11-15] | 13 [11-16] | 0.063 | 0.967 |
| FiO2, % | 90 [80-100] | 70 [60-80] | 60 [50-80] | 70 [60-90] | **< 0.01** | **< 0.01** |
| PaCO_2_, mmHg | 50 [45-58] | 49 [43-58] | 48 [42-56] | 48 [42-56] | **0.012** | 0.115 |
| PaO_2_, mmHg | 77 [66-90] | 102 [86-126] | 98 [84-117] | 88 [72-110] | **< 0.01** | 0.111 |
| PEEP, cmH_2_O | 12 [10-14] | 12 [10-14] | 12 [10-14] | 12 [10-14] | **0.025** | 0.417 |
| PaO_2_/FiO_2_, mmHg | 89 [72-110] | 150 [121-196] | 162 [124-221] | 135 [98-184] | **< 0.01** | **< 0.01** |
| Plateau pressure, cmH_2_O | 26 [23-28] | 25 [23-28] | 25 [22-28] | 26 [23-28] | 0.245 | 0.703 |
| Tidal volume, mL | 422 [374-467] | 420 [378-474] | 422 [380-474] | 429 [381-479] | 0.062 | 0.102 |
| Tidal volume/PBW, mL/kg | 6.34 [5.87-6.93] | 6.33 [5.86-7] | 6.39 [5.9-7.12] | 6.44 [5.9-7.17] | 0.055 | 0.101 |
| pH | 7.37 [7.32-7.42] | 7.38 [7.32-7.42] | 7.4 [7.34-7.44] | 7.4 [7.34-7.45] | **< 0.01** | **0.013** |

Data are presented as median [interquartile range]. FiO_2_ = Fraction of inspired oxygen, PaCO_2_ = Partial pressure of carbon dioxide in arterial, PaO_2_ = Partial pressure of dioxide in arterial blood, PEEP = Positive End Expiratory Pressure, PBW = Predicted Body Weight, PP = Prone positioning, SP = Supine positioning. Bold formatting of p value indicates a statistically significant value.

| **Variables** | **Before PP** | **At 16 hours of PP** | **End of PP** | **After SP** | **p for evolution between before PP and after SP** | **p for evolution between 16 hours of PP and end of PP** |
| --- | --- | --- | --- | --- | --- | --- |

**SUPPLEMENTARY TABLE S3 –** Ventilatory parameters during the first proning session (n = 81 sessions)

| Dynamic respiratory system compliance, mL/cmH_2_O | 34 [27-43] | 35 [28-44] | 35 [28-45] | 36 [28-45] | 0.122 | 0.512 |
| --- | --- | --- | --- | --- | --- | --- |
| Driving pressure, cmH_2_O | 12 [10-14] | 12 [10-14] | 12 [10-14] | 12 [10-14] | 0.456 | 0.949 |
| FiO_2_, % | 100 [80-100] | 70 [60-80] | 60 [50-80] | 70 [50-90] | **< 0.01** | **< 0.01** |
| PaCO_2_, mmHg | 47 [41-55] | 45 [40-51] | 45 [40-50] | 46 [41-51] | 0.14 | 0.897 |
| PaO_2_, mmHg | 73 [62-83] | 101 [86-120] | 96 [83-115] | 84 [73-115] | **< 0.01** | 0.368 |
| PEEP, cmH_2_O | 12 [10-13] | 12 [10-14] | 12 [10-14] | 12 [10-14] | **< 0.01** | 0.443 |
| PaO_2_/FiO_2_, mmHg | 78 [67-100] | 145 [110-214] | 162 [119-225] | 131 [98-202] | **< 0.01** | **0.016** |
| Plateau pressure, cmH_2_O | 24 [22-26] | 24 [22-27] | 23 [22-27] | 25 [22-28] | 0.658 | 0.606 |
| Tidal volume, mL | 419 [376-451] | 420 [382-456] | 420 [380-464] | 426 [386-463] | **0.032** | **0.048** |
| Tidal volume/PBW, mL/kg | 6.19 [5.8-6.85] | 6.32 [5.88-6.8] | 6.39 [5.92-7.02] | 6.46 [5.9-7.09] | 0.071 | 0.112 |
| pH | 7.36 [7.31-7.41] | 7.39 [7.34-7.43] | 7.41 [7.35-7.43] | 7.39 [7.35-7.43] | **< 0.01** | **0.027** |

Data are presented as median [interquartile range]. FiO_2_ = Fraction of inspired oxygen, PaCO_2_ = Partial pressure of carbon dioxide in arterial blood, PaO_2_ = Partial pressure of dioxide in arterial blood, PEEP = Positive End Expiratory Pressure, PBW = Predicted Body Weight, PP = Prone positioning, SP = Supine positioning. Bold formatting of p value indicates a statistically significant value.
